# A Mobile Education and Social Support Group Intervention for Improving Postpartum Health in Northern India: Development and Usability Study

**DOI:** 10.2196/34087

**Published:** 2022-06-29

**Authors:** Alison M El Ayadi, Mona Duggal, Rashmi Bagga, Pushpendra Singh, Vijay Kumar, Alka Ahuja, Ankita Kankaria, Darshan Hosapatna Basavarajappa, Jasmeet Kaur, Preetika Sharma, Swati Gupta, Ruchita S Pendse, Laura Weil, Dallas Swendeman, Nadia G Diamond-Smith

**Affiliations:** 1 Department of Obstetrics, Gynecology and Reproductive Sciences University of California, San Francisco San Francisco, CA United States; 2 Department of Epidemiology and Biostatistics University of California, San Francisco San Francisco, CA United States; 3 Postgraduate Institute of Medical Education & Research Chandigarh India; 4 Department of Obstetrics & Gynaecology Postgraduate Institute of Medical Education & Research Chandigarh India; 5 Department of Computer Science & Engineering Indraprastha Institute of Information Technology Delhi New Delhi India; 6 Survival for Women and Children Foundation Panchkula India; 7 Department of Community and Family Medicine All India Institute of Medical Sciences Bathinda India; 8 School of Medicine Stanford University Palo Alto, CA United States; 9 Department of Psychiatry and Biobehavioral Sciences University of California, Los Angeles Los Angeles, CA United States

**Keywords:** mHealth, group care, postpartum, postnatal, antenatal, India, pilot, mobile phone

## Abstract

**Background:**

Structural and cultural barriers limit Indian women’s access to adequate postnatal care and support despite their importance for maternal and neonatal health. Targeted postnatal education and support through a mobile health intervention may improve postnatal recovery, neonatal care practices, nutritional status, knowledge and care seeking, and mental health.

**Objective:**

We sought to understand the feasibility and acceptability of our first pilot phase, a flexible 6-week postnatal mobile health intervention delivered to 3 groups of women in Punjab, India, and adapt our intervention for our next pilot phase, which will formally assess intervention feasibility, acceptability, and preliminary efficacy.

**Methods:**

Our intervention prototype was designed to deliver culturally tailored educational programming via a provider-moderated, voice- and text-based group approach to connect new mothers with a social support group of other new mothers, increase their health-related communication with providers, and refer them to care needed. We targeted deployment using feature phones to include participants from diverse socioeconomic groups. We held moderated group calls weekly, disseminated educational audios, and created SMS text messaging groups. We varied content delivery, group discussion participation, and chat moderation. Three groups of postpartum women from Punjab were recruited for the pilot through community health workers. Sociodemographic data were collected at baseline. Intervention feasibility and acceptability were assessed through weekly participant check-ins (N=29), weekly moderator reports, structured end-line in-depth interviews among a subgroup of participants (15/29, 52%), and back-end technology data.

**Results:**

The participants were aged 24 to 28 years and 1 to 3 months postpartum. Of the 29 participants, 17 (59%) had their own phones. Half of the participants (14/29, 48%) attended ≥3 of the 6 calls; the main barriers were childcare and household responsibilities and network or phone issues. Most participants were very satisfied with the intervention (16/19, 84%) and found the educational content (20/20, 100%) and group discussions (17/20, 85%) very useful. The participants used the SMS text messaging chat, particularly when facilitator-moderated. Sustaining participation and fostering group interactions was limited by technological and sociocultural challenges.

**Conclusions:**

The intervention was considered generally feasible and acceptable, and protocol adjustments were identified to improve intervention delivery and engagement. To address technological issues, we engaged a cloud-based service provider for group calls and an interactive voice response service provider for educational recordings and developed a smartphone app for the participants. We seek to overcome sociocultural challenges through new strategies for increasing group engagement, including targeting midlevel female community health care providers as moderators. Our second pilot will assess intervention feasibility, acceptability, and preliminary effectiveness at 6 months. Ultimately, we seek to support the health and well-being of postpartum women and their infants in South Asia and beyond through the development of efficient, acceptable, and effective intervention strategies.

## Introduction

### Background

Against the backdrop of persistent rural poverty in Northern India, modest gains in maternal health have been made over the past decade, with maternal mortality decreasing from 251 to 113 maternal deaths per 100,000 live births [1,2]. Despite this progress, further improvements in maternal health are needed for India to achieve the United Nations Sustainable Development Goal target of <70 maternal deaths per 100,000 live births by 2030 [3], including a broader focus on achieving the full continuum of perinatal care. The COVID-19 pandemic has significantly impeded global progress in achieving Sustainable Development Goal targets by exacerbating maternal and perinatal adversity, with significantly greater impacts seen among low- and middle-income countries such as India compared with higher-income settings [4].

Postnatal care and support are important contributors to optimizing maternal and neonatal health and well-being and a critical component of the full continuum of perinatal care [5]. High-quality postnatal care and social support (ie, emotional, instrumental, and informational) have been associated with reduced maternal and neonatal mortality [5,6] and increased maternal engagement in behaviors promoting newborn (eg, exclusive breastfeeding and child immunization) and maternal (eg, postnatal adoption of family planning) health [7,8]. Acknowledging the important role of postnatal care, the Government of India recommends that women receive 3 postnatal visits from community health workers [1]. However, despite India’s broad community-based maternal health programming, a significant drop in the continuum of peripartum care occurs in the postnatal period [9,10]. Postnatal care achievement is generally low across the country, with 65% of Indian women receiving at least one health check within 2 days of birth, ranging from 48% to 80% across wealth quintiles, and full postnatal care achievement being anecdotally low [1].

Substantial structural and cultural barriers prevent new mothers from attending postnatal care at facilities or other locations that may be far from their homes, particularly in India. Common logistical challenges are exacerbated in India by rural geographic distances, cultural and linguistic barriers to care, women’s practice of postnatal seclusion, and generally low levels of mobility in marriage, all culminating in reduced care access [11-13]. Further intergenerational and gender-based hierarchical roles structure decision-making in Indian households, particularly for couples living in extended-family households where decision-making may be largely outside the hands of women [14]. Within this context, novel methods for improving women’s access to the full peripartum continuum of health care are required.

Mobile phone–based health approaches (mobile health [mHealth]) offer innovative opportunities to overcome logistical barriers to postnatal care access. Despite women’s physical mobility limitations in this setting, 88% of Indian households nationally own a mobile phone, including 72% of women [1,15]. Theory-informed mobile support and education groups have positively affected [16] maternal and neonatal health outcomes [17-20], and mHealth approaches to pregnancy care suggest high acceptability, promising results, and high cost-effectiveness [21,22]. However, preventive mHealth models extending care postnatally are sparse [23-25]. Group-participatory learning and action models (eg, women’s groups) are another promising model for improving postnatal care given their efficient approach, acceptability, and effectiveness in improving maternal and neonatal health indicators [26,27]. Such models provide efficient health education, strengthen social support networks, and influence social norms [28]. Social support [29,30] reduces postnatal depression and anxiety [31,32] and improves breastfeeding maintenance [33,34] and women’s empowerment [35]. CenteringPregnancy and other group-based supportive prenatal care models affect exclusive breastfeeding, contraceptive uptake, depression, and immunization postnatally [36-40]. Extending group care postnatally is an innovative and promising approach for improving health outcomes for both mothers and neonates through targeted education and support for women in the postnatal period and referral to in-person care.

### Objectives

This study describes the development and pilot results of a targeted education and support intervention for postnatal Indian women where they can interact with providers and other new mothers, named *Maa Shishu Swasthya Sahayak Samooh* (maternal and child health support group; *MeSSSSage*). With the goal of optimizing the feasibility and acceptability of our intervention, we implemented a 2-phase developmental process to pilot test and refine the intervention functions, processes, and delivery platform. In this paper, we report on the results of pilot phase 1, a flexible 6-week postnatal mHealth intervention delivered to 3 groups of women in Punjab, India, and describe the integration of these results into optimizing the intervention for our phase 2 pilot study, in which we will seek to assess the feasibility and acceptability of the full 6-month intervention more formally.

## Methods

### Study Context

Maternal and child health indicators in Punjab, Northern India, show some improvement, but persistent challenges remain, suggesting that innovative strategies are needed for improving health outcomes and ensuring high-quality care. Across the perinatal continuum of care, 68.5% of new mothers receive at least four antenatal care visits and 90.5% have facility births, but only 40% of women receive any postnatal care [1]. Many Punjabi women of reproductive age are anemic (53.5%), and anemia rates are higher among postpartum women [41]. Modern contraceptive use is 63% overall [42], yet postpartum uptake of contraceptives is lower. A study found that only 30% of Punjab women adopted a contraceptive method postpartum, most often condoms, with 16% having an unmet need [43]. Postpartum mental health remains understudied; however, a recent systematic review of postpartum depression in India reported a pooled estimate of 22% and identified lack of social support as a primary risk factor [44]. Most children aged 12 to 23 months (89.1%) are fully immunized; however, appropriate diarrheal disease and acute respiratory infection management in children aged <5 years is inadequate (range 26.7%-90.3%) [42]. Only 5% of children aged <2 years receive adequate nutrition, and approximately one-fifth of children aged <5 years are stunted (25.7%) or underweight (21.6%) [42]. Disparities in maternal and child health indicators exist according to rurality of residence and socioeconomic status [42].

### Intervention Prototype Development

**Figure 1:**
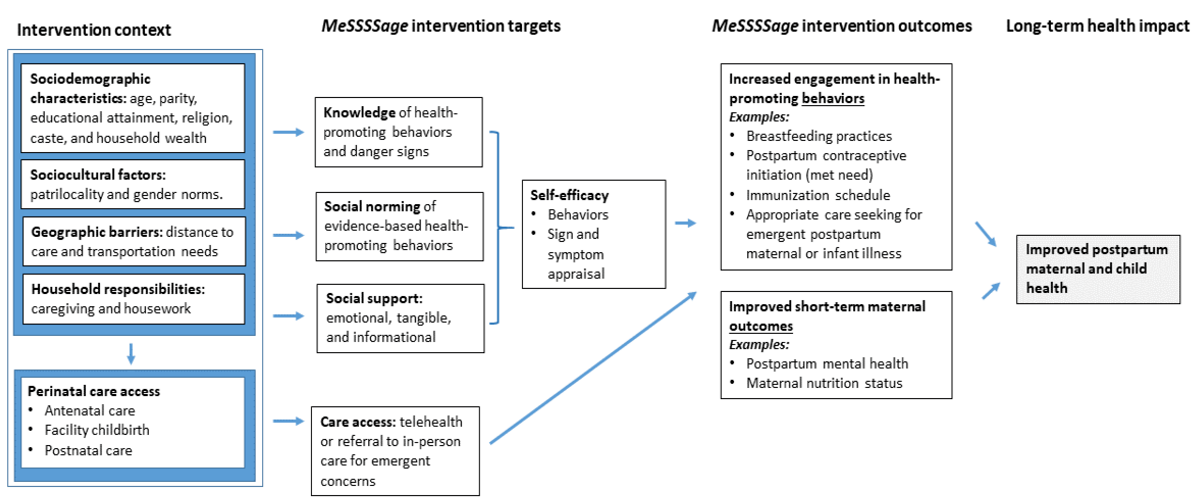
Conceptual framework of Maa Shishu Swasthya Sahayak Samooh (maternal and child health support group; *MeSSSSage*) intervention context, targets, outcomes, and anticipated health impacts.

The initial *MeSSSSage* prototype was developed by a team of Indian and US-based maternal health clinicians, researchers, and human-centered technology design experts. The prototype was based on the interdisciplinary literature, early-stage formative needs assessment research with postnatal women, and local and international care guidelines. *MeSSSSage* was designed to overcome the prevalent structural and cultural barriers to postnatal maternal and neonatal health care in this setting by delivering culturally tailored educational programming via a provider-moderated, voice- and text-based group approach to connect mothers with a social support group of other new mothers, increase their health-related communication with providers, and refer them in a timely and appropriate manner to care. Our conceptual framework (Figure 1) outlines a summary model of the factors identified in our formative work that influence perinatal care access, knowledge, health behaviors, and health outcomes, and the pathways that the *MeSSSSage* intervention has been designed to disrupt. The intervention is registered at ClinicalTrials.gov (NCT04636398).

### Intervention Conceptual Framework

**Figure 2:**
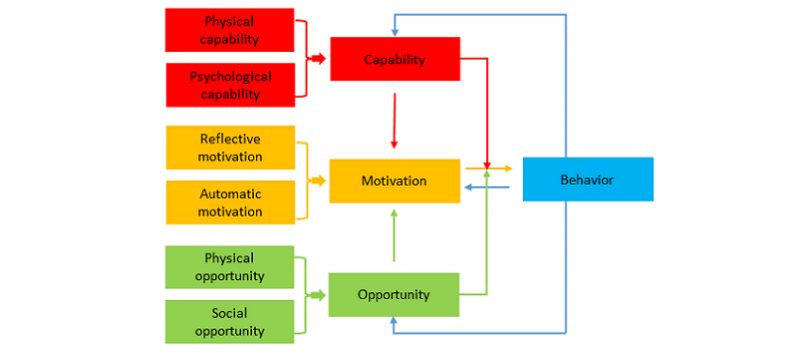
Capability, Opportunity, Motivation-Behavior model of behavior change [45,46].

Capability, Opportunity, Motivation-Behavior is a comprehensive framework for behavior change intervention design that posits that behaviors (B) are the result of interactions between capability (C), opportunity (O), and motivation (M; Figure 2) [45,46]. The *MeSSSSage* intervention builds on the Capability, Opportunity, Motivation-Behavior framework to affect health behaviors as follows: (1) the mHealth approach allows women to participate in the intervention from their homes to reduce the impact of social, sociocultural, geographic, and logistical barriers, increasing *physical and social opportunity*; (2) the educational content is designed to increase knowledge of important maternal and child health–promoting behaviors, increasing *psychological capability* and *reflective motivation*; and (3) the social support and social norming component is designed to increase *reflective and automatic motivation.* We hypothesize that providing targeted education and support to women in the postnatal period through a mobile social network where they can interact with providers and other new mothers will improve their knowledge of health-promoting behaviors and their parental self-efficacy and empowerment. By appropriately identifying infant and maternal danger signs and promoting timely care seeking for routine and emergency visits (including referrals), we seek to reduce both maternal and infant mortality and morbidity. Furthermore, mHealth group support could help encourage and sustain exclusive breastfeeding and improve uptake of postnatal family planning and childhood vaccination, which positively affects maternal and child health in both the short and long term.

### Intervention Educational Content

Maternal and newborn educational content was developed through a review of Indian and international clinical guidelines, consultation with experienced local maternal and child health providers, and formative research to identify health education needs among our target population. Topics were organized according to anticipated maternal and neonatal information needs by postpartum week (Table 1). The discussion facilitated during the group calls focused on ensuring women’s understanding of the content and addressing questions. An obstetrician or gynecologist and a pediatrician advised on emergent health concerns and referred women for in-person care.

**Table 1:** Maternal and newborn educational content, Maa Shishu Swasthya Sahayak Samooh (maternal and child health support group) pilot intervention, phase 1.

Intervention week	Maternal educational topics	Newborn educational topics
1	Breastfeeding, postpartum hygiene, and self-care	Infant danger signs
2	Diet and nutrition and maternal danger signs	Temperature regulation
3	Mental health and COVID-19 precautions	Neonatal care
4	Emotional support and family planning overview	Massage
5	Family planning methods and postnatal care visits	Developmental milestones
6	General health issues and use of mobile health technology for health improvement	COVID-19 precautions

### Technology Development

For this phase 1 pilot, our team prioritized deployment across feature (nonsmart) phones for inclusivity of diverse groups of women, including those of lower socioeconomic status. We developed 2 platforms for group call implementation and the dissemination of educational audios using FreeSWITCH open-source, voice-over IP servers (Multimedia Appendix 1). Group calls initiated from the server were free for intervention participants; however, accessing educational audio recordings outside of the weekly calls incurred call charges to the participants. All intervention content was in the Punjabi language.

A mobile SMS text messaging group that included participants and moderators was created for each intervention group using WhatsApp (Meta Platforms). This SMS text messaging group was used to share group call reminders and educational material and to asynchronously provide additional education and support to the intervention participants. The participants were invited to use the WhatsApp group to share stories or experiences with their group members and to ask questions of other participants and moderators. Some discussions were facilitated by the group moderator per our intervention testing plan (Table 2). The WhatsApp groups were accessible to all participants with smartphones and many participants with feature phones.

**Table 2:** Intervention delivery approaches by domain, Maa Shishu Swasthya Sahayak Samooh (maternal and child health support group) pilot intervention, phase 1.

Week and domain		Group 1	Group 2	Group 3
**Week 1**				
	Educational component delivery^a^	Live	Live	Recorded
	Participation^b^	Talk	Hand	Talk
	SMS text messaging group moderation^c^	Not moderated	Moderated	Moderated
**Week 2**				
	Educational component delivery^a^	Live	Live	Recorded
	Participation^b^	Talk	Hand	Talk
	SMS text messaging group moderation^c^	Not moderated	Moderated	Moderated
**Week 3**				
	Educational component delivery^a^	Live	Live	Recorded
	Participation^b^	Hand	Talk	Hand
	SMS text messaging group moderation^c^	Not moderated	Moderated	Moderated
**Week 4**				
	Educational component delivery^a^	Recorded	Recorded	Live
	Participation^b^	Hand	Talk	Hand
	SMS text messaging group moderation^c^	Moderated	Not moderated	Not moderated
**Week 5**				
	Educational component delivery^a^	Recorded	Recorded	Live
	Participation^b^	Talk	Hand	Talk
	SMS text messaging group moderation^c^	Moderated	Not moderated	Not moderated
**Week 6**				
	Educational component delivery^a^	Recorded	Recorded	Live
	Participation^b^	Talk	Hand	Talk
	SMS text messaging group moderation^c^	Moderated	Not moderated	Not moderated

^a^For *live*, educational messages are delivered by the moderator during the group call, intermixing the material with open time for questions. For *recorded*, prerecorded educational messages are accessed via phone before the group call, with the full group call used for discussion.

^b^For *hand*, the women have to raise their hands to talk, with the moderator unmuting them to allow for their participation. For *talk*, the women can talk freely.

^c^For *moderated*, a group moderator facilitates group participation through consistent use of prompts in the group. For *not moderated*, the SMS text messaging group is introduced to the women for their own use with no researcher facilitation.

### Intervention Delivery

The pilot intervention was delivered over a 6-week period (December 2020 to January 2021). This was between the first and second COVID-19 surges in India and, thus, although some restrictions were in place, COVID-19 rates were low, and care access was not seriously limited. Weekly group calls were planned for approximately 30 to 40 minutes and scheduled at the same time each week. SMS text message reminders were sent to the participants 1 day beforehand. Participation in the WhatsApp chat group was encouraged. Group calls were moderated by 3 research team members: a moderator to handle group dynamics (AA), an obstetrician or gynecologist (RB or DHB), or a pediatrician (VK).

### Varying Delivery Approaches

To determine the best structure and understand how to optimize engagement, we varied three delivery approaches to assess the women’s reception of delivery of educational content, participation in group discussion, and format of the SMS text messaging–based chat platform (Table 2): (1) the educational content being provided live by the moderator versus through a recorded message delivered through interactive voice response before the group call, (2) participation in calls occurring via the participants *raising their hand* by pressing 1 on their keypad to indicate that they would like to speak and being unmuted by the technical moderator versus all participants being unmuted for the full call, and (3) the WhatsApp SMS text messaging group being moderated by a provider through consistent use of prompts in the group versus not being moderated (both groups could text questions in the chat for the provider to answer). Each group experienced each of these modalities for half of the intervention.

### Study Site and Participants

The study was conducted within Block Boothgarh, Mohali District, Punjab state, Northern India. Block Boothgarh comprises 129 villages with a total population of 5000 served by 16 health subcenters, each with 1 auxiliary nurse midwife (ANM) who maintains a consolidated perinatal care register. Mobile phone ownership per local care registers is high among households (90%) and women (50%).

Study participants included women residing in the study area who had given birth within the previous 3 months. Further inclusion criteria were being aged <40 years and having a live neonate with birth weight >1500 g. The exclusion criteria were maternal complications during or after childbirth warranting hospital stay and continued medical care at the facility, stillbirth, twins, significant birth defects, inability to provide informed consent, and lack of phone access if unwilling to accept a phone from the study team. If the eligible participants did not have a personal phone, they were offered a study phone.

Potential participants were identified with assistance from local ANMs. Women who met the study inclusion criteria based on ANM records were contacted telephonically by a study researcher who further screened them and explained the study procedures in detail, including the risks and benefits of participation. The women confirmed their consent verbally. Where requested, assent was obtained from the husband or another family member in alignment with local norms. At study enrollment, the women were oriented to the developmental stage of the intervention. A total of 100 women were contacted by phone, and 41 (41%) were considered eligible based on further screening. Of these 41 women, 29 (71%) agreed to participate, and 12 (29%) declined. The 29 women were sequentially enrolled in 3 groups of 7 (24%), 10 (34%), and 12 (41%) based on their child’s birth date. We sought to limit variation in birth date across groups (+2 weeks to −2 weeks) to ensure that the women were at similar postnatal stages but maintained some heterogeneity in group size for understanding group size dynamics.

### Data Collection

At enrollment, sociodemographic data were collected from the participants on age, parity, village, contact number, mobile phone ownership (none, individually owned, or shared with another household member), mobile phone type (smart vs feature phone), and willingness to accept a mobile phone from the study team if they did not have their own mobile phone. Intervention feasibility and acceptability were assessed through tracking attendance, brief weekly individual check-in calls among most participants (20/29, 69%), weekly moderator reports, structured end-line in-depth interviews (IDIs) among a subset of the intervention participants (15/29, 52%), and back-end data from the mobile technologies used.

Weekly check-in calls conducted with group members were used primarily for individual problem solving and facilitative follow-up and collected data on attendance challenges, intervention acceptability (ie, level of satisfaction with the overall intervention and the intervention educational, group discussion, and SMS text messaging chat components), experience with that week’s call, technical issues, perspectives on the usefulness of the educational content and group discussion, need for additional material, perspectives on changes made in intervention delivery if relevant (Table 1), and any other recommendations. In addition, after each group call, moderators noted successes, challenges, technical disruptions, significant interactions, and quality of engagement between participants and between participants and moderators and captured the participants’ questions.

End-line IDIs were conducted among a subset of intervention participants (15/29, 52%) to capture their overall intervention satisfaction; their experiences with the program; the usefulness of the educational content, group discussion, and SMS text messaging group; their perspectives on participation and group dynamics; their perspectives on content and call logistics (eg, call structure, participants, and moderator preferences); and their recommendations for improving the intervention. Structured questions using a Likert-type response scale were included on overall satisfaction, usefulness of the intervention components (educational content, group discussion, and SMS text messaging group), likelihood of recommending the group, and level of connectedness.

Back-end data collected from the calls included the list of listeners, the list and time stamp of the audios played, the list of participants who *raised their hand to talk*, the number of times each participant was redialed, and the audio recording of each group call. From this, we captured the number of participants per session and the number of participants who engaged with the health worker and each other. We also gathered data from WhatsApp on SMS text messaging engagement, including posts, views, and topics. For the prerecorded audios, we collected the call duration (how long the women listened to them) and which audio recordings were accessed (topic).

### Ethics Approval

Due permissions were obtained from the Indian Council of Medical Research and senior health authorities of the Government of Punjab and Mission Director, National Health Mission, India. The study protocol was approved by the University of California, San Francisco Institutional Review Board (19-299723); the Ethics Committee of the Post Graduate Institute of Medical Education and Research (IEC-03/2020-1567); the Collaborative Research Committee of the Post Graduate Institute of Medical Education and Research (79/30-Edu-13/1089-90); and the Indian Council of Medical Research (ID 2020-9576).

## Results

### Participant Characteristics

A total of 29 women participated in our pilot intervention (Table 3). The women ranged in age from 24 to 28 years and had all given birth within the previous 1 to 3 months for the first or second time. Educational attainment varied, with 10% (3/29) of the women being illiterate and 10% (3/29) having a postgraduate education. Infants were generally aged 3 to 4 weeks at first call. All participants (29/29, 100%) had some phone access, either a personally owned phone or a shared household phone; 59% (17/29) of the participants had a personal phone. The 41% (12/29) of participants with no personal phones were offered mobile phones and SIM cards by the research team. Of these 12 participants, 7 (58%) accepted the phones, and 5 (42%) continued to participate from shared household phones. The group members did not know each other before joining the study. Data from end-line IDI participants identified that most of the women (12/15, 80%) lived in multigenerational households with in-laws. Most end-line IDI participants (11/15, 73%) reported limited phone use and some experience with WhatsApp, Facebook, and YouTube.

**Table 3:** Participant characteristics, Maa Shishu Swasthya Sahayak Samooh (maternal and child health support group) pilot intervention, phase 1 (N=29).

Characteristic		Values
Age (years), median (IQR)		25 (24-28)
Parity, median (IQR)		1 (1-2)
**Educational attainment, n (%)**		
	Illiterate	3 (10)
	Primary	2 (7)
	Higher primary	4 (14)
	Secondary	3 (10)
	Senior secondary	11 (38)
	Graduation	3 (10)
	Postgraduation education	3 (10)
**Baby’s age (weeks) at enrollment, n (%)**		
	1 to 2	6 (21)
	3 to 4	18 (62)
	5 to 6	5 (17)
**Mobile network, n (%)**		
	Airtel	9 (31)
	Idea	6 (21)
	Jio	9 (31)
	BSNL^a^	3 (10)
	Vodafone	2 (7)
Phone owned personally, n (%)		17 (59)
Smartphone access, n (%)		28 (97)
From Punjab, n (%)		22 (76)

^a^BSNL: Bharat Sanchar Nigam Limited.

### Attendance

Approximately half of the 29 participants (14/29, 48%) attended ≥3 of the 6 weekly group calls. Attendance across the 6 sessions was highest in the first few calls at 55% (16/29) for the first call and 69% (20/29) for the second call, but declined over time, with between 41% (12/29) and 48% (14/29) attending group calls 3 to 5 and 28% (8/29) attending the final weekly group call. The last group call attended was call 1 for 3% (1/29) of the participants, call 2 for 10% (3/29) of the participants, call 3 for 7% (2/29) of the participants, call 4 for 7% (2/29) of the participants, and call 5 for 34% (10/29) of the participants, and 28% (8/29) of the participants attended through the sixth group call.

Reported barriers to call attendance included infant care or other household responsibilities, visitors, network issues, and phone access. Some participants did not have their phones with them all the time, had them switched off, or participated using their husbands’ phones, which were not always accessible (particularly during the workday). For example, a participant stated that “*...*there was lot of work at home. My mother-in-law is sick already and with two kids, I barely get time for anything*.*”

### Intervention Acceptability

The participants reported high acceptability of the pilot intervention. Across the weekly check-ins, all of the reporting participants reached reported that they were very (16/19, 84%) or somewhat satisfied (3/19, 16%) and that they would be very likely to recommend the intervention to others (19/19, 100%). Among the end-line IDI respondents, 80% (12/15) were very satisfied, and 20% (3/15) were somewhat satisfied. Feedback on the intervention elements is detailed in the following sections.

### Educational Content

Across the weekly check-ins, all of the reporting respondents (20/20, 100%) reported that the educational content was very useful. All end-line IDI respondents reported the educational content to be very useful (9/15, 60%) or somewhat useful (6/15, 40%). The participants shared their appreciation that content on both maternal and infant concerns was included and felt that the educational content met their needs. However, a couple of participants mentioned already being familiar with most of the educational content. Of the 15 end-line IDI respondents, 13 (87%) preferred receiving educational content in the form of audio recordings, mentioning that the information was always accessible and could be saved and listened to again and forwarded to others.

### Group Discussion

All participants indicated that the group discussion was very useful (17/20, 85%) or somewhat useful (3/20, 15%) in the weekly check-ins. End-line IDI respondents largely found the group discussion to be very useful (13/15, 87%), with other participants reporting that it was somewhat useful (1/15, 7%) or feeling neutral (1/15, 7%). The participants appreciated the open discussion and the opportunity for everyone to ask questions. A woman shared that “everybody got time to discuss their queries and got their answers.” Most participants appreciated the way the group orientation of the discussion allowed all participants to learn from the questions and experiences of their peers that were brought up on the call and believed that the time allocated for discussion was adequate. Some women suggested that the opportunity for one-to-one discussion could make women more comfortable with certain questions as “there are few topics on which we hesitate to talk in front of other people” and would be helpful for urgent queries. However, others felt that, if this were the case, that material should be brought back to the group for learning; for example, “...we can talk to each other. So [if] one-to-one talk can be done, it will be helpful. Sometimes women don’t open up to doctors easily so when we talk to each other, we get to know that we are going through same thing. It will be better.” The moderators noted that, although participant comfort with asking questions of the moderators improved over time, there was some reluctance to interact directly with each other with little unprompted engagement. Despite this, 79% (15/19) of the weekly check-in respondents reported feeling very or somewhat connected to other members of their group.

### Text Messaging Chat

Back-end data of the WhatsApp groups showed that the number of communications ranged by group, from 59 in group 1 to 114 in group 3. Most were SMS text messages (74/114, 64.9% to 47/59, 80% across the groups), with some audio messages (8/59, 14% to 21/92, 22.8% and 26/114, 22.8% across the groups) and some images and videos (6/92, 7% to 12/114, 10.5% across the groups). The proportion of communications initiated by the participants (vs moderators) ranged from 21% (19/92) to 43% (49/114) across the groups. Educational needs were voiced in 9% (4/47) to 7.9% (9/114) of SMS text messages across the groups and included questions on both maternal (ie, pain after cesarean section, bleeding, dietary advice, and family planning) and infant (ie, spitting up, rashes, upper respiratory infections, fever after vaccination, baby massage, and crying) health concerns. Social messages were voiced in 4% (4/92) to 15.8% (18/114) of the SMS text messages, including wishing group members a happy new year and solstice.

End-line IDI participants reported infrequent chat group use but appreciated the questions asked via both SMS text message and audio message and the moderator’s rapid response. The few who reported using the SMS text messaging group largely felt comfortable; however, a couple of women reported privacy and security concerns. Several women reported checking the SMS text messaging chat once per day when their husbands returned from work if they were participating in the intervention on their husbands’ phones.

### Call Structure

The overall structure of the calls was considered to be good by the end-line participants, with most suggesting that the calls should last approximately 30 minutes. The participants largely reported a preference for the health education content to be prerecorded so that it could be listened to on their own time before the call where the group could discuss the content.

### Call Participants

A couple of the women’s husbands joined the call, seemed engaged, and asked questions, although it was unclear whether this affected the other women’s comfort on the call. When asked in end-line IDIs, there was some variation in perspectives regarding whether husbands or other family members should be allowed on calls, with 67% (10/15) of respondents stating that only women should be on the call and the other 33% (5/15) stating that it would be fine if the husbands joined.

### Group Moderator Preferences

Although the participants were satisfied with the pilot group moderators, most participants (14/15, 93%) felt that a physician moderator would be preferred for such an intervention. Only 3% (1/29) of the participants felt that a community health worker (accredited social health activist) would be acceptable as a moderator. Of the 29 participants, 2 (7%) felt that a female physician would be preferable, but others said that it would not make a difference.

### Technical Challenges

Beyond the aforementioned challenges to attendance, call challenges reported by both participants and moderators included difficulty hearing voices and recordings. The dialing platform used for the group calls had audible calling sounds when dialing and redialing if the participants dropped off the call. These sounds caused disturbances in the ongoing live discussion and the educational audio, thus interrupting the presentation. The group calls also suffered from significant network issues, which resulted in dropped calls and unclear audio. In addition, children were sometimes heard crying in the background when the women were unmuted and asking questions.

### Other Lessons Learned

Moderators noted that fostering connection between participants felt challenging and suggested that beginning the group calls in late pregnancy could help the women form relationships before the busy early postnatal period. In addition, moderators felt that including women who were both primi- and multiparous in the intervention added depth to the conversation as multiparous women could provide additional support and mentorship to primiparous women.

### Design Decisions for Pilot Phase 2

The aforementioned findings and other considerations relevant to design decisions for formalizing the next iteration of the *MeSSSSage* intervention were reviewed and discussed by the research team. Each consideration is presented in Table 4 by domain (participants, intervention, and participation and engagement), including the options considered, decisions made, and summary rationale.

**Table 4:** Intervention design for Maa Shishu Swasthya Sahayak Samooh (maternal and child health support group) pilot intervention, phase 2.

Domain and attributes			Options		Decision		Summary rationale
**Participants**							
	Selection criteria: parity	Primiparous only vs both primiparous and multiparous		Prioritize primiparous women but allow some multiparous women		The intervention is likely to be more impactful for first-time parents, but optimally facilitating discussion requires a large enough group. Multiparous participants may play an important role in sharing experiences and facilitating group discussion because of their experience.	
	Selection criteria: birth mode	Separate groups by birth mode (vaginal and cesarean) vs keeping them together		Maintain all participants together regardless of birth mode		Women with cesarean birth have unique early postpartum recovery needs; however, with our decision to start the intervention antenatally to increase group cohesiveness, further reshuffling of groups based on birth mode would be detrimental. An extra session for cesarean births could be added.	
	Selection criteria: allowing other individuals to participate	Limit to women only vs allowing or encouraging others to attend; consider attendee gender		Discourage but not prohibit others besides women from attending		Prioritizing privacy and confidentiality is key. Various family members attended some group sessions and perspectives were mixed; some were open to others attending; however, approximately half reported discomfort with non–group members on the calls, particularly men. Moderators felt that husband attendees asked important questions that contributed to the group discussion. An extra session that includes husbands or family members could be added.	
**Intervention**							
	Timing of recruitment and intervention	Recruit and begin intervention antenatally vs postpartum or recruit women antenatally and begin intervention in the early postpartum period		Recruit antenatally (28-32 weeks); hold 2 antenatal sessions (at approximately 32 weeks and 36 weeks) with 6 months of weekly postpartum sessions starting at 39 weeks; keep groups together regardless of birth date		Recruiting participants and initiating the groups antenatally provides the opportunity for participants to build rapport and relationships before the postpartum period, which may be more hectic. Inclusion of 2 groups antenatally allows for the promotion of health-promoting antenatal and birth practices. These benefits outweigh the disadvantages of the wider range in birth date and infant age possible within each group. A small number of participants will be expected to leave the groups because of severe maternal or neonatal complications.	
	Group size	12 to 20 participants per group		Target 20 participants per group		Pilot group size ranged from 7 to 12. We were initially concerned that too many people per group would overwhelm the sessions. However, attrition reduced the number of participants per session. Larger groups can accommodate any reductions in group attendance associated with antenatal recruitment and attrition.	
	Frequency of group calls	Weekly, twice per week, every 2 weeks, monthly		2 antenatal sessions and weekly postpartum sessions		Participants endorsed group calls twice per week; however, we anticipated feasibility concerns and eventual attrition with this intervention burden. A predictable weekly schedule routinizes the calls. We prioritized some antenatal engagement to build group familiarity before birth and meet some antenatal health education needs.	
	Group call length	Between 20 to 60 minutes		Target 20 minutes, allow up to 60 minutes		Previous educational information dissemination helps calls focus on group discussion. The women seemed to drop-off with longer calls.	
	Distribution of educational information	Live sharing of educational material vs dissemination of recorded content before the group call		Distribute educational material before group calls through short audio recordings; calls will include brief educational highlights		Educational material on the call and via recordings was acceptable; however, participant drop-off was higher on longer calls. Prerecorded audios allow for educational content to be accessed via an app (smartphone users) and via an IVR^a^ system (feature phone users). Moving this education outside of the group discussion shortens the meetings, which may reduce barriers to full attendance and transitions within the group discussion.	
	Moderators	Physicians vs midlevel health professionals		Community health officers, including nurse midwives or community physicians, with some specialist support		Midlevel health professionals balance training and skills required for the moderator role and resource use, and they typically have high technological literacy. Use of these individuals as primary moderators with access to specialists for complex concerns is likely to be a scalable approach. Specialists will be featured on a few calls.	
	Connecting to the group call	Have network call out vs participant calls in to network or IVR		Network calls out to participants		This approach does not require the women to initiate the call, poses the lowest cost to women (no cost), and it does not exclude feature phone users. We have identified an alternative dialing platform that minimizes the disruptions observed in pilot phase 1 (ie, dialing sounds and other audio issues).	
	SMS text messaging chat group	All vs some groups with SMS text messaging chat group		All participants with WhatsApp-capable phones will be added to the SMS text messaging chat group.		Some women did not have their own phone and were unwilling to accept a phone from the research team. This could challenge participation in this component of the intervention for these women, and participation in the SMS text messaging chat group using a shared phone results in privacy concerns. Text chat groups will be facilitated by intervention moderators.	
	Phone type (overall and across groups)	Include only smartphones vs only feature phones vs both		Include both, mix groups where appropriate		Although access to smartphones is increasing rapidly across India, vulnerable women who may benefit more from intervention participation are less likely to have smartphones. Maintaining both phone types in the intervention is more complicated but more scalable.	
**Participation and engagement**							
	Building relationships and participation	Earlier recruitment; use of introductions and icebreakers; facilitation of WhatsApp group for better group cohesion		Highlight privacy of the group; incorporate icebreakers into each session; recruit earlier (during antenatal care)		Increasing group engagement is a top priority of our intervention with our strong focus on social support. Improving participation and group dynamics requires a multi-pronged approach.	
	Privacy	Use names vs anonymous; privacy reminders; disclose other listeners; allow for call recording or not; provide earphones		Use the women’s first names and ask how they want to be addressed; do not allow participants to record discussions without permission		There is an inherent tension between respecting privacy and confidentiality and building relationships within the group. Our moderators will prioritize the comfort level of each group’s participants. Where participants are interested in recording group discussions for sharing with others, this will only be allowed with permission from the full group.	
	Participation mechanism	Raise hand via pressing a number vs being unmuted all the time; calling on participants vs natural flow		First focus on promoting spontaneous discussion via unmuting all and then move to calling on people as needed		Ensuring that group discussions participation is easy for our participants while limiting external noise that may distract or make it hard to hear. We will integrate more structured opportunities for building group cohesion to promote comfort with discussion.	

^a^IVR: interactive voice response.

## Discussion

### Principal Findings

Overall, *MeSSSSage* proved to be a highly acceptable approach to provide information and social support to this hard-to-reach and undersupported population—postnatal women in rural India. However, sustaining participation over time and fostering group interactions was difficult because of 2 primary challenges. The first was technological, where our preliminary mHealth platform had many issues related to network connection and audible dialing of the back-end program. The second was due to sociocultural factors related to women’s comfort and cultural norms around sharing personal experiences with other women and interacting with physicians given the hierarchical social structures. These 2 challenges are common in mHealth [47] and are addressable through continuous improvement of the design of *MeSSSSage*; the goal of this phase 1 pilot was to identify such challenges.

To address technology-related challenges, we have outsourced the management of group calls to a cloud-based service provider. Given global server access, such providers are able to circumvent the reliability issues we experienced with local network providers by supplying a panel to hold internet-based calling. In our preliminary tests with this service, none of the previously occurring bothersome network and technological issues arose. We also engaged a reputed interactive voice response service provider for prerecorded educational content dissemination and developed an app for smartphone users, enabling improved access to educational content for both feature and smartphone users.

To address the second set of challenges as a result of interpersonal interactions, we are adapting our intervention model in several ways. First, instead of recruiting women immediately after giving birth, we will recruit them during late pregnancy and hold 2 group sessions prenatally. This will allow women within the same group to get to know each other before they give birth, before they are managing a newborn infant and recovering from childbirth. We will also integrate more icebreakers, get-to-know-you games, and discussion starters into each session, which should help group members get to know each other and become more comfortable interacting. Finally, although feedback on husbands’ participation was mixed, with some women supporting their participation and others feeling uncomfortable with their involvement, we have decided to not allow husbands to participate to increase the comfort of all participants. Although we realize that we cannot forcibly exclude husbands given the mobile nature of the intervention and because houses may be small and phones shared, we will encourage husbands to allow the women to participate on their own given the personal nature of pre- and postnatal concerns. Other recent research on gender dynamics around phone use in India has highlighted the role of husbands as gatekeepers to women’s phone use, especially when phones are shared [48]. We will carefully monitor intervention implementation to ensure that exclusion of husbands from the intervention does not lead to increases in intimate partner violence or other relationship issues and explore future opportunities for formal engagement of husbands in similar interventions. Finally, although many women said that they liked the sessions being moderated by physicians, the moderators in the pilot and other team members felt that this might hinder women’s comfort talking because of the culture of respect around hierarchy [49]. We also hypothesized that part of the reason why the women said that they liked having physicians as moderators was that that was the only type of moderator they were exposed to and because of the potential for social desirability bias given that the moderators and other research team members were all from the same institution. Therefore, we will incorporate midlevel health care providers as regular intervention moderators but will invite obstetrician-gynecologists and pediatricians to attend certain sessions to respond to participant questions and provide advice. Our selection of community health officers for this role is supported by their primary roles within Ayushman Bharat, the flagship scheme of the Government of India to achieve comprehensive universal health coverage, and the National Digital Health Mission [50,51]. We will also ensure that all moderators are women.

### Conclusions

The findings of this study support the *MeSSSSage* intervention’s unique combination of group-based care and mHealth approaches for improving postnatal health in a geography where significant cultural and logistical barriers exist to completing the continuum of perinatal care as an innovative and promising approach for improving both maternal and neonatal health. The importance of mHealth interventions such as ours, which can successfully provide information and social support without in-person interactions, was also emphasized by the COVID-19 pandemic context in which we piloted our intervention. The revisions to overcome the primary challenges and other considerations will be implemented in the next pilot test of the *MeSSSSage* intervention. Our second intervention pilot will incorporate continued assessment of intervention feasibility and acceptability as well as preliminary assessment of effectiveness in preparation for robust effectiveness testing in subsequent research, as indicated. Ultimately, we seek to support the health and well-being of postpartum women and their infants in South Asia and beyond through the development of efficient, acceptable, and effective intervention strategies.

## Appendix

Multimedia Appendix 1 Technology development.

## Abbreviations

ANM: auxiliary nurse midwife
IDI: in-depth interview
MeSSSSage: Maa Shishu Swasthya Sahayak Samooh (maternal and child health support group)
mHealth: mobile health
