# Supplementary material for: A Mobile Education and Social Support Group Intervention for Improving Postpartum Health in Northern India: Development and Usability Study
Source: JMIR Form Res. 2022 Jun 29;6(6):e34087. doi: 10.2196/34087 (PMC9280461; doi:10.2196/34087)
Supplement: Multimedia Appendix 1 [file formative_v6i6e34087_app1.docx]

**Multimedia Appendix 1**

***Technology development***

For the phase 1 pilot, our team focused on deployment for feature (non-smart) phones in order to increase participation across diverse groups of women, including those of lower socioeconomic status. We developed two platforms for group call implementation and the dissemination of educational audios (supplemental text). Both platforms employed FreeSWITCH, an open-source voice over internet protocol (VOIP) server to manage the telephony aspects which is managed by a Python script that connects to the FreeSWITCH server through the Event Socket Layer (ESL) and issues commands on the server to execute the required functionalities. The group call platform was used to initiate the group call, view the list of participants, play/pause/stop informational audios, mute/unmute participants, view participants who wanted to to ask a question, and redial participants who dropped from the call. The platform used a GSM Service to translate VOIP service into GSM. The technical moderator ran the python script and issued commands to the FreeSWITCH server to conduct the group call through the interface provided by the script according to the format of the group call. This platform was completely free for listeners to use. The educational audio dissemination platform followed a similar mechanism in reverse, using GM Service to translate GSM into VOIP. A python script always ran in the background and the FreeSWITCH server was continuously available for calls from the women. Women’s calls went directly to the FreeSWITCH server, which welcomed the caller and presented them with a choice to listen to audios on maternal or newborn healthcare topics by inputting a certain digit. Under each topic, the caller was presented with the option of seven topical audios that could be selected through pressing digits on their phone. All intervention content was in Punjabi language. After listening to one audio completely, the caller was again presented with the option to listen to the audios under the chosen topic. On receiving no response from the caller for two minutes, the call was disconnected. The pilot platform was capable of handling only one call at a time. In this platform, the call charge was on the caller. A WhatsApp group was created with the participants and moderators of group calls as a mode of dissemination of group call schedules and to provide additional education and support. This was presented to the participants as a place where they could share their stories, experiences and concerns with the group for learning as well as ask questions of others, both participants and moderators. Per our intervention testing protocol, group moderators facilitated participation in the WhatsApp group through asking questions.
